# Degree of Bacterial Contamination of Mobile Phone and Computer Keyboard Surfaces and Efficacy of Disinfection with Chlorhexidine Digluconate and Triclosan to Its Reduction

**DOI:** 10.3390/ijerph15102238

**Published:** 2018-10-12

**Authors:** Jana Koscova, Zuzana Hurnikova, Juraj Pistl

**Affiliations:** 1 Department of Microbiology and Immunology, Institute of Microbiology and Gnotobiology, University of Veterinary Medicine and Pharmacy in Košice, Komenského 73, 041 81 Košice, Slovakia; juraj.pistl@uvlf.sk; 2 Institute of Parasitology, Slovak Academy of Sciences, Hlinkova 3, 040 01 Košice, Slovakia; hurnikz@saske.sk

**Keywords:** mobile phone, computer keyboard, bacteria, disinfection, reduction of contamination

## Abstract

The main aim of our study was to verify the effectiveness of simple disinfection using wet wipes for reduction of microbial contamination of mobile phones and computer keyboards. Bacteriological swabs were taken before and after disinfection with disinfectant wipes with active ingredients chlorhexidine digluconate and triclosan. The incidence and type of microorganisms isolated before and after disinfection was evaluated; the difference was expressed as percentage of contamination reduction. Our results confirmed the high degree of surface contamination with bacteria, some of which are opportunistic pathogens for humans. Before the process of disinfection, on both surfaces, mobile phones, and computer keyboards, the common skin commensal bacteria like coagulase-negative staphylococci were diagnosed most frequently. On the keyboards, species of the genus *Bacillus* and representatives of the family Enterobacteriaceae were abundant. The potentially pathogenic species were represented by *Staphylococcus aureus*. Cultivation of swabs performed 5 min after disinfection and subsequent calculation of the reduction of contamination have shown that simple wiping with antibacterial wet wipe led to a significant reduction of microbial contamination of surfaces, with effect ranging from 36.8 to 100%.

## 1. Introduction

Microbial standards in hygiene are necessary for a healthy life. People often believe that microbes are only present in research labs or in hospitals and clinics and thus they have a misleading feeling of security in other places. Lack of knowledge about where germs occur could be the cause of health problems. In fact, 80% of infections are spread through hand contact with hands or other objects [1]. Bacteria are found almost everywhere in air, water, soil, food, and in plants and animals organisms, including humans. It is generally acknowledged that inanimate objects can also carry microorganisms originating from the surrounding environment. Predominantly Gram-positive cocci (*Staphylococcus* spp., *Micrococcus* spp.), but also spore-forming rods (*Bacillus* spp.) or Gram-negative bacteria, can be transmitted through devices like mobile phones or computer keyboards [2]. These attached microorganisms have a potential to be transferred to food or human body, where the growth of bacteria may continue. Furthermore, formation of biofilm by one bacterial agent can affect the survival of other pathogens on the same surface [3]. Once deposited on surfaces, many infectious agents can survive for extended periods unless they are eliminated by disinfection or sterilisation procedures [4]. Depending on environmental conditions, pathogens may remain infectious on surfaces for weeks after being contaminated [5].

Mobile phone usage has a personal character, being attached to the close proximity of parts of the body such as the face, ears, nose, lips, and hands, which are the most common infection gateways. Transferred microorganisms can, especially in people with suppressed immune system, cause opportunistic infections and mild to chronic disease.

Computer keyboards are among the most commonly used user interfaces. The majority of keyboards have over 101 individual keys, which makes it difficult and time consuming to clean. This is often the reason why most owners do not clean and disinfect the keyboard.

Today, mobile phones have become one of the most indispensable accessories for professional and social life. In addition to the standard voice function of a telephone, mobile phones can support many additional services such as SMS (Short Message Service) for text messaging, email, pocket switching for access to the Internet, and MMS (Multimedia Messaging Service) for sending and receiving photos and video [3]. Although mobile phones are usually stored in bags or pockets, they are handled frequently and held close to the face [6]. Mobile phones can spread infectious diseases by their frequent contact with hands [7] and they have also been reported to be a reservoir for microorganisms [8].

Mobile phones are also in close relationship to nosocomial infections, they may act as a mobile reservoir for microbial pathogens [9]. The use of cell phones often occurs in hospitals, by patients, visitors, and health care workers, and this is one environment where hospital-associated (nosocomial) infections are most prevalent [10]. One study came to a result that pathogenic bacteria are present on approximately 40% of mobile phones belonging to patients in a hospital and on approximately 20% of mobile phones belonging to hospital staff [3].

As well as mobile phones, computer keyboards have been also implicated as a potential reservoir for infectious agents [11]. Given that computers are not routinely disinfected, the opportunity for the transmission of contaminating microorganisms is potentially great. The computerߣs keyboard and also mouse have a very dynamic environment. In general, the bacteria that live on our skin, fingernails, hands, and anywhere the hands have been are likely to transfer new bacteria over to the keyboard. Especially, in a place where there is a lot of people moving in and out, such as a hospital, school or office, there is likely to be a good number of people that are sick, and through them come the new bacteria that will eventually settle on the keyboard through the air or from physical contact. Inadequately performed hand hygiene and not disinfected surfaces are two reasons why the computer keys could be the sources of microbial contamination, consequently resulting in indirect transmission of potential pathogens [12]. Eating above computer keyboards is also one of the causes of bacterial contamination. Spills can wind up on and between the keys, and the food deposits encourage the growth of millions of bacteria. Dust can trap moisture and enable any bacteria that are already on the keyboard to flourish [13].

Studies dealing with cell phone or keyboard contamination are mainly focused on HAI (hospital-acquired infections) and transmission of nosocomial pathogens [14,15]. The present study was undertaken to evaluate the bacteriological contamination of mobile phones and computer keyboards and their susceptibility patterns to commonly used disinfectant wipes with active ingredients chlorhexidine digluconate and triclosan. This work was not conducted in a hospital, rather the samples were taken from devices of people working in microbiology lab and teachers. The aim of this study was to show that mobile devices present potential risk of infection not only in hospitals. Mobile devices and electronic keyboards can carry pathogens that can be harmful to human beings. While many believe pathogen transmission is only harmful in healthcare settings, many people lack knowledge that the transmission of these harmful bacteria can occur in everyday life activities and underrate the disinfection of these devices. Using disinfectant wipes once daily for mobile phones and keyboards can decrease the probability of contamination and spreading of bacterial pathogens through these devices.

## 2. Materials and Methods

### 2.1. Sample Collecting

Swabs from the investigated surfaces of 25 mobile phones and 25 computer keyboards were collected using disposable sterile cotton swabs moistened with sterile saline. For sampling, the devices of employees of University of Veterinary Medicine and Pharmacy in Košice (Department of Microbiology and Immunology) were taken on a voluntary basis. These people are working at University, the swabs were taken from teachers as well as from laboratory workers who are working in microbiology laboratories. Samples were collected by thorough rotating a cotton swab on the surface and the back of the mobile phone, including keypad, touch screen, and the both sides of the phone. Subsequently, the entire telephone was disinfected using disposable, commercially available disinfectant wipes with active ingredients chlorhexidine digluconate and triclosan, and after 5 min the control smear was repeated in the same manner with new sterile swab. Swabs from the keyboard were carried out the same way, with thoroughly wiping out the space between the keys and individual keys. Control swab was performed also 5 min post disinfection.

Prior the collection of swabs, the respondents–users of mobile phones and computer keyboards completed a questionnaire with basic data on the use and cleaning of the device.

Hands are a critical component of the human microbiome. Proposed model for the hand as a critical vector in microbiome dynamics was designed by Edmonds-Wilson et al. [16]. Part of this model that deals with spread of bacteria through fomites was used to guide our study.

### 2.2. Isolation

For primary isolation of bacteria withdrawn from the swab, blood agar, MacConkey agar (HiMedia, India), and Sabouraud agar (HiMedia, India) were used. The blood agar was prepared as Tryptone Soya Agar (HiMedia, India) mixed with 5% of defibrinated sheep erythrocytes that served for detection of haemolytic activity of bacteria. Blood agar and MacConkey agar plates were incubated aerobically at 37 °C for 24 h, Sabouraud agar plates were incubated in aerobic conditions at 25 °C for 48–72 h. After cultivation, the colonies were identified by their characteristic appearance on cultivation media (including haemolysis and typical growth characteristics).

In addition to the culture examinations, obtained pure bacterial cultures were diagnosed by means of microscopic examination. To observe motility of bacteria, native preparations were prepared by method of compressed drop. Gram staining was used for differentiation between Gram-positive and Gram-negative bacteria, and also for determination of the size, shape, and specific arrangement of observed pure solitary colonies.

### 2.3. Identification of Isolates

Isolated bacteria were consequently confirmed by the pattern of biochemical reactions. Biochemical tests evaluated the ability of the bacteria to produce the enzyme catalase and oxidase, as well as the ability of the members from the family Enterobacteriaceae to ferment the sugar series. Test for indol detection, H_2_S production, methyl red test (MRT), and detection of urease activity were also carried out (ENTEROtest 24 N, Erba-Lachema, Brünn, Czech Republic). In the differential diagnosis of *Staphylococcus* spp., results of STAPHYtest (Erba-Lachema, Czech Republic) and the production of coagulase in rabbit plasma (Bio-Rad, Hercules, CA, USA) as an important factor in the virulence of pathogenic staphylococci were evaluated.

**Figure 1:**
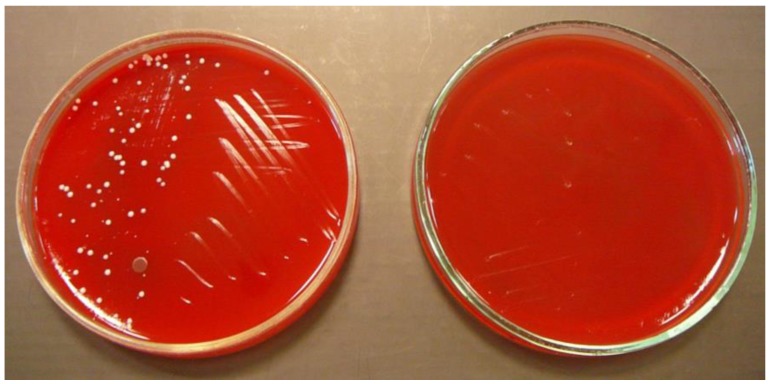
Blood agar with bacteria isolated from mobile phones before (**left**) and after (**right**) disinfection.

**Figure 2:**
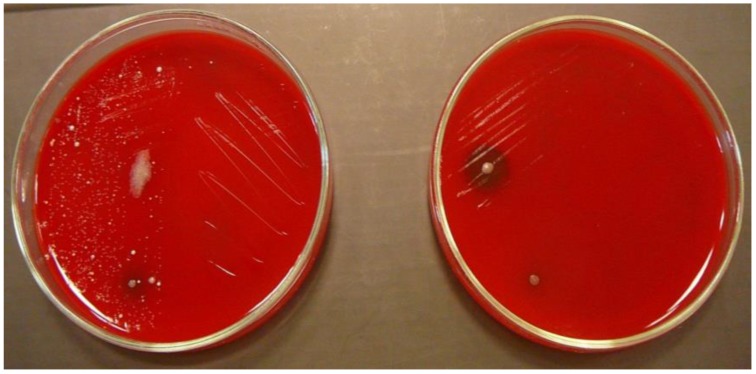
Blood agar with bacteria isolated from keyboards before (**left**) and after (**right**) disinfection.

In the experiment, the number of isolated microorganisms was determined before disinfection and compared with the number of microorganisms isolated after disinfection (Figure 1 and Figure 2); the difference was expressed as a percentage of contamination reduction on tested surfaces after disinfection.

### 2.4. Statistical Analysis

The relation of microbial contamination of device surfaces from users habits expressed in the questionnaires was statistically evaluated by means of Chi-square test using Microsoft Office Excel 2013. The difference was considered statistically significant if the achieved level of significance (*p*) was less than 0.05.

## 3. Results

Out of 25 mobile phones under investigation, 92% were contaminated with bacteria. Most frequently staphylococci were isolated (Table 1). In samples before disinfection, *S. aureus* (20%) and CoNS—coagulase negative staphylococci (76%)—were identified. On mobile phone surfaces, bacilli and micrococci were also present (both 36%). The lowest numbers were represented by enteric bacteria (12%). The identification carried out based on biochemical tests in test tubes, and the use of ENTEROtest 24 N, revealed the presence of coliform bacteria as *Escherichia coli* (100%), *Enterobacter cloaceae* (66%), *Klebsiella pneumoniae* (33%), and *Citrobacter freundii* (33%).

The computer keyboards were most contaminated with bacilli and staphylococci (Table 2). In total, 96% of keyboards were contaminated. Except for these bacteria, other Gram-positive bacteria were also detected, mainly members of genera *Micrococcus* and *Streptococcus*. Gram-negative bacteria were represented by members from the family Enterobacteriaceae–*Escherichia coli* (50%), *Enterobacter cloaceae* (37.5%), and *Citrobacter freundii* (25%). Yeasts and fungi were also detected, but without species specification.

After disinfection, in all isolated bacteria taken from sampled surfaces, a reduction of microbial contamination was observed. Simple disinfection using antimicrobial wipe was followed by elimination of the number of bacteria to zero in 60.9% of mobile phones and 16.7% of keyboards, which is a statistically significant difference (*p* ˂ 0.001).

Post disinfection, the numbers of bacteria on mobile phone surfaces decreased radically—by 60% in case of *S. aureus* and almost identically by 63.2% in case of CoNS, the decrease in bacilli and micrococci was even 88.9%, and number of enteric bacteria was reduced by 100%. Microbial contamination of keyboards was reduced after disinfection by 100% in the case of *Streptococcus* spp., yeasts, and fungi; on the contrary, the reduction of *Bacillus* spp. was only 31.8%.

The evaluation of questionnaire survey did not demonstrate a significant relationship between the monitored user interface parameters (age, type, and method of wearing, use and type of enclosure, hand washing after using the toilet) and contamination levels. Thirty-two per cent of respondents clean their mobile with a frequency of 1–4 times per a month, and 52% of respondents treat the keyboard on average 1–2 times a month. However, the difference in the microbial contamination of devices that are regularly cleaned and those that are not cleaned was not statistically significant.

Our research confirmed the efficacy of a simple cleaning with disinfectant wet wipe with chlorhexidine digluconate and triclosan in reduction of bacterial contamination.

## 4. Discussion

The emergence of bacterial infection in humans is increasing, similarly the occurrence of community infections is increasing [17]. One of the main causes of epidemics obtained from the environment and nosocomial infections is the bio-contamination of surfaces of various items and equipment used by the public. Contaminated surfaces become fomites transmitting infectious organisms between inanimate and living objects, and in the infectious chain serve as the reservoir for pathogens, from which they spread further through transfer via hands [18]. According to the results by Weber [19], the subsequent transmission ability of the pathogen depends on the interaction of the host, pathogenic agent, and the environment.

An important role is played by the transfer of germs to the surface of the object, the subject on hand to mouth and hands, which are often the gateway for infection [20,21]. Reynolds et al. [22] used an invisible fluorescent marker to track public contamination of surfaces and have found that as many as 86% of individuals are brought to the contamination of the hands of the home. Some bacteria can thereby be infectious even in very low doses and can survive for several hours or days in the biofilm formed on smooth surfaces.

We investigated the occurrence of microorganisms on everyday objects—mobile phones and computer keyboards. The incidence of bacteria was detected on 92% mobiles and 96% of keyboards, with a mixed flora of Gram-negative and Gram-positive, and potentially pathogenic or non-pathogenic bacteria. The results of our work confirmed that 92% of mobile phones were examined microbial contaminated, usually by *Staphylococcus epidermidis*. These bacteria are part of the physiological microbiota of the skin and mucous membranes as epiphytes and commensal bacteria. Coagulase-negative staphylococci were the most prevalent bacteria also in the study of Pal et al. [23], almost 81%. In addition, we established the presence of representatives of the genera *Bacillus* and *Micrococcus* (both 36%). Enteric bacteria (12%) and *S. aureus* (20%) were present in the lowest numbers on mobile phone surfaces. These bacteria, under certain circumstances, mainly in immunosuppressed persons, may cause purulent infections in humans. Comparable results were published by Singh et al. [24], who indicated the most common isolates from mobiles were the coagulase negative staphylococci (39.78%), compared to our results (76%); 16% for *S. aureus*, while our results showed 20%; and bacteria belonging to *Bacillus* spp., *Micrococcus* spp., *Pseudomonas* spp., and *Acinetobacter* spp. Significant contamination of mobile phones (99%) was also found by Bhat et al. [25], with the finding of *S. aureus*, *E. coli*, *Klebsiella pneumoniae*, *Acinetobacter* spp., *Pseudomonas aeruginosa*. Ilusanya et al. [26] detected 100% contamination with 50% prevalence of *S. aureus*, *Streptococcus faecium* (34%), *Bacillus cereus* (32%), *E. coli* (26%), and *Micrococcus luteus* (10%) on mobile phone surfaces. The presence of different types of microorganisms on mobile phones has also been confirmed by Ulger et al. [27] and Soto et al. [28], who found that contamination of mobile phones most commonly occurs through the hands, bags, cases, pockets, but also the environment and food residue. Higher temperature and humidity generated by mobile phone support bacterial growth and biofilm formation on the surface of the device. Depending on the environmental conditions, the pathogens in the biofilm may persist in infectious state for several weeks [29]. Except for bacterial contamination, *Candida* spp. [30] or even viruses [31] can also be transmitted by the mentioned devices.

Ilusanya et al. [26] confirmed that, through hands to food transfer, a contaminated cell phone can infect the food and induce infection. E.g., if using the phone in the toilet, despite consecutive hand washing, food might be contaminated during consumption by the medium of the mobile phone. It follows that the phone should not be taken to the toilet, bathroom, or put on polluted surfaces.

In study of Kurli R. et al. [32], matrix-assisted laser desorption ionization time of flight (MALDI-TOF) mass spectrometry was used to generate protein profiles for 527 isolates obtained from cellular phones by swabs. A dendrogram was constructed based on the protein profiles of the remaining isolates, to group 112 isolates under 39 different proteotypes. The representative strains of each group were selected for 16S rRNA gene and ITS (Internal Transcribed Spacer) region sequencing-based identification. *Staphylococcus*, *Bacillus*, *Micrococcus*, and *Pseudomonas* were the most frequently encountered bacteria, and *Candida*, *Aspergillus*, *Aureobasidium*, and *Cryptococcus* were in case of fungi. At species-level, the prevalence of *Micrococcus luteus*, *Staphylococcus hominis*, *Staphylococcus epidermidis*, *Staphylococcus arlettae*, *Bacillus subtilis*, and *Candida parapsilosis* was observed, most of these species are commensal microorganisms of human skin.

Our research confirmed that keyboards are contaminated with more heterogeneous spectrum of microorganisms as compared to mobile phones, as evidenced by the presence of yeasts and fungi that were not detected on the phones. Compared with mobile phones, also the detection of bacilli was more than 50% higher. Bacilli are bacteria that occur ubiquitously are mostly saprophytic, feeding on dead organic matter. The presence and persistence of these bacteria in the environment is related to the ability to form highly resistant spores that protect bacterial genome from the adverse effects of the external environment. For their occurrence on keyboards, the fact that 60% of respondents to our survey eats upon the device may be responsible.

Despite the fact that 52% of addressed persons clean the keyboard more or less regularly, 88% of them were contaminated with bacilli. Also notable is the lower reduction in the number of bacilli after disinfection compared with mobile phones. While on the phone, one rub with disinfectant damp cloth reduced the incidence of bacilli by 88.9%, but on keyboards the reduction was less emphatic (31.8%). Several authors have confirmed that keyboards are heavily microbial polluted and represent a frequent source of infection in hospitals and schools [33,34,35].

In our research, the user’s habits of respondents in relation to the occurrence of microorganisms on device surfaces were also evaluated. Our results point to an interesting paradox. While 32% reported cleaning of the phone, and up to 52% of them reported cleaning of the keyboard, a statistically significant difference in contamination between treated and untreated devices was not ascertained.

Following standardly performed disinfection with disinfection wipes that contain active ingredients such chlorhexidine digluconate and triclosan, the reduction of contamination to zero was achieved in 60.9% of mobile phones, but only in 16.7% of keyboards, which is a statistically, highly significant difference (*p* ˂ 0.001). This may be due to the spaces between the keys are difficult to clean, and in particular the accumulation of dust and organic substrate in these sites may favour the survival of bacteria. It is proven that on polluted surfaces more microorganisms occur, which underlines the need for removal of dirt and dust to reduce contamination. The advantages provide keyboards with solid surface, touch buttons etc. [36].

Our research demonstrates that microbial contamination of mobile phones and computer keyboards is frequent and the most common organisms are skin commensals. The presence of potentially pathogenic bacteria such as *S. aureus*, Gram-positive bacilli, and enteric bacteria represent a risk of infection especially for immunocompromised people.

Periodic cleaning of mobile phones with disinfectants or hand cleaning detergents, as well as frequent hand-washing, has been encouraged as a mean of curtailing any potential disease transmission [4,11]. Keyboards can be safely and successfully decontaminated with disinfectants, such are phenol-based wipes with alkaline detergents or 70% isopropyl-alcohol [37]. Methylated spirit can also be used for these purposes [38]. Many of the recommended disinfectants are based on chlorine, alcohol, phenol, or ammonium that can cause skin reactions or functional or cosmetic damage to object surfaces.

## 5. Conclusions

In the present study, it was confirmed that using commercially available antibacterial wet wipes can reduce the presence of microorganisms on devices of daily use like mobile phones or computer keyboards. The reduction of bacterial contamination after disinfection achieved even 100% in the case of enteric bacteria on mobile phone surface, and also 100% in case of *Staphylococcus aureus* or *Streptococcus* spp. on the surface of computer keyboards. After disinfection, no yeasts or moulds were present on the devices.

Since disinfection of computer keyboards is rather difficult, precautions such as hand washing and good hygiene practice among users is advocated to prevent the possibility these devices as vehicles of pathogen transmission. Basics of proper standards of hygiene, respiratory etiquette, and hand washing should be part of regular education and training. These should also include methods for decontamination and disinfection of computers and phones, especially those that are available to the general public.

## Figures and Tables

**Table 1:** Percentage evaluation of bacteria isolated from mobile phone surfaces (*n* = 25) before and after disinfection and reduction of contamination.

Organisms	Before Disinfection	After Disinfection	Reduction of the Contamination
*Staphylococcus aureus*	5 (20%)	2 (8%)	60.0%
CoNS	19 (76%)	7 (28%)	63.2%
*Bacillus* spp.	9 (36%)	1 (4%)	88.9%
*Micrococcus* spp.	9 (36%)	1 (4%)	88.9%
Enteric bacteria	3 (12%)	0 (0%)	100%

Legend: CoNS—coagulase negative staphylococci.

**Table 2:** Percentage evaluation of microorganisms isolated from computer keyboard surfaces (*n* = 25) before and after disinfection and reduction of contamination.

Organisms	Before Disinfection	After Disinfection	Reduction of the Contamination
*Staphylococcus aureus*	1 (4%)	0 (0%)	100%
CoNS	20 (80%)	8 (32%)	60.0%
*Bacillus* spp.	22 (88%)	15 (60%)	31.8%
*Micrococcus* spp.	5 (20%)	2 (8%)	60.0%
*Streptococcus* spp.	3 (12%)	0 (0%)	100%
Enteric bacteria	16 (64%)	8 (32%)	50.0%
Yeasts	2 (8%)	0 (0%)	100%
Moulds	3 (12%)	0 (0%)	100%

Legend: CoNS—coagulase negative staphylococci.
